# Chronic Kidney Disease: Role of Diet for a Reduction in the Severity of the Disease

**DOI:** 10.3390/nu13093277

**Published:** 2021-09-19

**Authors:** Tania Naber, Sharad Purohit

**Affiliations:** 1 Department of Interdisciplinary Research, College of Allied Health Sciences, Augusta University, Augusta, GA 30912, USA; tnaber@augusta.edu; 2 Department of Undergraduate Health Professionals, College of Allied Health Sciences, Augusta University, Augusta, GA 30912, USA; 3 Department of Gynecology and Obstetrics, Medical College of Georgia, Augusta University, Augusta, GA 30912, USA; 4 Center for Biotechnology and Genomic Medicine, Augusta University, Augusta, GA 30912, USA

**Keywords:** diabetes, chronic kidney disease, proteinuria, diabetes, inflammation, diet, nutrition, plant-based foods, medical nutrition therapy

## Abstract

Chronic kidney disease affects ~37 million adults in the US, and it is often undiagnosed due to a lack of apparent symptoms in early stages. Chronic kidney disease (CKD) interferes with the body’s physiological and biological mechanisms, such as fluid electrolyte and pH balance, blood pressure regulation, excretion of toxins and waste, vitamin D metabolism, and hormonal regulation. Many CKD patients are at risk of hyperkalemia, hyperphosphatemia, chronic metabolic acidosis, bone deterioration, blood pressure abnormalities, and edema. These risks may be minimized, and the disease’s progression may be slowed through careful monitoring of protein, phosphorus, potassium, sodium, and calcium, relieving symptoms experienced by CKD patients. In this review, the current Kidney Disease Outcomes Quality Initiative (KDOQI) recommendations are highlighted, reflecting the 2020 update, including explanations for the pathophysiology behind the recommendations. The Dietary Approaches to Stop Hypertension, the Mediterranean diet, and the whole foods plant-based diet are currently being examined for their potential role in delaying CKD progression. Biological explanations for why the whole foods plant-based diet may benefit CKD patients compared to diets that include animal products are examined. Strong evidence continues to support the importance of diet meeting the daily requirement in the prevention and progression of kidney disease, and medical nutrition therapy with a registered dietitian is a critical aspect in medical intervention for CKD.

## 1. Introduction

The kidneys control many biological mechanisms such as fluid, electrolyte, pH balance, blood pressure, excretion of toxins and waste, vitamin D metabolism, and hormone synthesis. About thirty-seven million US adults are estimated to have chronic kidney disease (CKD), which is more than one in seven [1] Even more astonishing, nine in ten adults do not know they have the disease, and half of the adults with little kidney function who are not on dialysis are unaware they have CKD [1]. Chronic kidney disease often goes undiagnosed due to a lack of apparent symptoms in early stages. An estimated 94% with mild to moderate decline in renal function and about 48% of individuals with severe renal dysfunction go undiagnosed [2].

**Figure 1:**
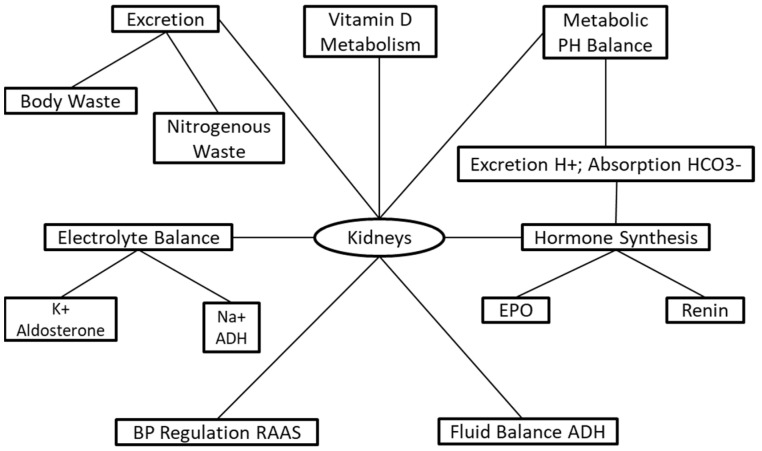
The physiological functions of kidneys.

The kidneys are responsible for a series of life-sustaining mechanisms (Figure 1). The primary functions of the kidneys are to sustain and maintain fluid and electrolyte and metabolic acid–base balance, which is accomplished through solute and fluid regulation, conservation of nutrients, and excretion of metabolic bodily waste [3]. The kidneys have endocrine and exocrine functions regulating and maintaining critical biological mechanisms in the body [4]. The exocrine functions involve fluid and electrolyte balance [5], acid–base regulation [6], and excretion of body waste [7] (Figure 1). The endocrine functions include the activation of vitamin D for the incorporation of calcium into bones [8], and hormone synthesis for the regulation of blood pressure and synthesis of red blood cells [8,9].

The National Kidney Foundation (NKF) defines CKD as either a decline in glomerular filtration rate (GFR) to <15 mL/min/1.73 m² or the presence of kidney damage persisting for at least three months [10]. The prevalence of diabetes and hypertension is growing exponentially, predicting that CKD will continue to rise [11]. CKD patients are at increased risks for other health conditions, including acute kidney injury (AKI), T2DM, and mortality [12]. Chronic kidney disease is nationally incorporated into health promotion and disease-prevention programs to reduce its prevalence [13]. The US Department of Health and Human Services Healthy People 2020 had a target goal to minimize CKD prevalence from 14.8% in 2001 to 13.3% by 2020 [14].

Medical nutrition therapy is imperative for CKD patients because it may slow the progression of the disease through careful monitoring of protein, calcium, phosphorus, potassium, and sodium [15], relieving symptoms experienced in CKD patients while not restricting too many nutrients that would put the patient at high risk for malnutrition [16]. This review covers CKD pathophysiology, the most current diet recommendations, and the mechanisms that may delay the progression of the disease. In addition, the mechanisms of the newly explored whole food plant-based diet (WFPBD) are explained for its possible advantages in CKD prevention and progression. We performed a literature search on PubMed using “medical nutrition therapy”, “chronic kidney disease”, “clinical trials”, and “outcomes of medical nutrition therapy in chronic kidney diseases” from January 2021 to May 2021. Published articles reporting clinical trials were selected for writing this review, and the information from these papers were incorporated as tables. To be included in this narrative review, the paper had to be a clinical trial on: (a) type of protein intake and its relevance to CKD, (b) maintaining calcium, phosphate, and vitamin D (VD) levels, and (c) electrolyte balance in CDK patients. All other articles were excluded. The main contribution in this review is to provide current clinical research to dieticians and physicians in a concise manner that introduces possibilities in acquiring an appropriate CKD diet that widens dietary variation by including foods with less nutrient bioavailability than animal products and additives. In addition, we provide points for future research needed, such as RCTs, which may produce data that may support the efficacy of a whole food plant-based diet on ameliorating CKD progression.

## 2. Medical Nutrition Therapy

The NKF published the first Kidney Disease Outcomes Quality Initiative (KDOQI), which is a set of nutritional guidelines for patients with end-stage renal disease in 1996 [17]. Since then, the KDOQI guidelines have gone through revisions and expanded to include nutrition recommendations for each stage of CKD, dialysis, and pre/post-kidney transplant [17,18]. Recommendations provided in this review are from the recent KDOQI Clinical Guideline for Nutrition in CKD: 2020 Update, which was developed with the Academy of Nutrition and Dietetics.

### 2.1. Protein and Renal Function

The federal Dietary Guidelines for Americans recommend an amount of 0.8 g/kg/body wt/d dietary protein intake for healthy adults [19]. Exceeding the recommended dietary allowance (RDA) may increase the risk of health complications even for healthy adults [19]. Protein intake recommendations for CKD patients are dependent on the stage of the disease, which is determined by declining GFR function [18].

The effects of high-protein diets (HPDs) on renal health have been investigated since the 1920s when rats given a HPD presented with increased kidney weight [20]. Data suggest that chronic protein intake (more than 1.2 g/kg/body weight/d) [21] leads to increased pressure and glomerular morphologic changes, resulting in renal dysfunction [22]. Glomerular hyperfiltration is defined as modifying renal hemodynamics through glomerular capillary hyperemia and increasing intraglomerular pressure [21]. HPDs induce glomerular hyperfiltration, hyperemia, and increased hydraulic pressure, resulting in vasodilation of the afferent arteriole [22]. HPDs contribute to progressive glomerular damage, which, combined with the renal deterioration from diseased kidneys may contribute to CKD progression. The Modification of Diet in Renal Disease (MDRD) was the largest RCT to examine the hypothesis that dietary protein restriction delays the progression of CKD [PMID 10541304]. The study found proteinuria to be one of the two strongest predictors in the rate of CKD progression in two studies [23]. Oba et al. collected 43 healthy (non-diseased) kidneys from live human donors to examine the effect of an HPD on the single-nephron GFR (SNGFR) [24]. This study concluded that an HPD might increase SNGFR and induce glomerular hyperfiltration; however, this study is unique by identifying that the analysis of human SNGFR is an exemplary parameter to alterations in renal hemodynamics at the single-nephron level [24]. The exact mechanism for renal hemodynamic responses to heightened protein intake is not yet understood [25].

Low-protein diets (LPDs) have been shown to improve hyperfiltration, reduce nitrogenous waste, and ease the renal workload by decreasing glomerular pressure [21]. Proteinuria declined by 20–50% in CKD patients who adhered to a LPD [26,27]. Although LPDs provide direct benefits to CKD patients, healthcare professionals are concerned about protein-energy malnutrition and protein-energy wasting (PEW) in CKD patients due to inadequate energy intake [26,27]. When determining the estimated energy requirements for CKD patients, 25–35 kcal/kg/body weight/d is recommended to maintain energy and nitrogen balance and avoid risk for malnutrition [16] (Table 1).

### 2.2. Very Low-Protein Diet

Low-protein diets and very low-protein diets (VLPD) (0.28–0.43 g/kg/body wt/d) may be achieved with nutrition supplementation with essential amino acids (EAAs) and ketoanalogues [27] to safeguard against PEW. The KDOQI guidelines recommend restricting protein to slow ESRD progression and improve quality of life (QoL) by reducing symptoms for metabolically stable patients [18]. The NKF defines metabolically stable as being absent from inflammatory or infectious diseases, poorly controlled diabetes, consumptive diseases, antibiotics or immunosuppressive medications, significant short-term loss of body weight, and no hospitalizations within two weeks [18,29]. For patients with CKD stage 3–5, a protein restriction providing 0.55–0.60 g/kg/body wt/d or a VLPD with supplementation with ketoacid analogues is recommended [18,29]. Diabetic adults with CKD 3–5 are recommended a protein diet providing 0.6–0.8 g/kg/body weight/d, and patients on maintenance hemodialysis (HD) or peritoneal dialysis (PD) with or without diabetes are recommended a protein intake providing 1.0–1.2 g/kg/body weight/d [20,30]. Diet modifications, such as reducing protein from heme sources and including plant-based proteins, protect against metabolic acidosis by lowering acid production; these effects are mostly seen with a VLPD (0.3–0.5 g/kg/body weight/d) with supplementation with ketoacid analogues [27,29]. Conservative reductions in protein intake as small as 0.1–0.2 g/kg/day have shown significant effects in preserving kidney function, hence slowing CKD progression [31]. A randomly controlled trial (RTC) reported a vegetarian (VLPD) (0.3 g/kg/body wt/d) supplemented with ketoanalogues compared with a standard LPD (0.6 g/kg/body wt/d) ameliorated kidney function decline over time and reduced the need for renal replacement therapy (RRT) [32].

An alternate protein source may be more beneficial to the patient’s health than restricting the amount of protein alone; the protein source may be of greater importance than the quantity [18,29]. Plant proteins are typically ingested along with fiber, phytonutrients, and antioxidants, although animal proteins are ingested along with saturated fat and cholesterol [2]; this may be one reason plant proteins are associated with a more vast decline in blood pressure compared to animal protein, as shown from the INTERMAP Study on micronutrients and macronutrients on blood pressure [33]. Additionally, animal protein is associated with decreased insulin sensitivity, increased reactive oxygen species (ROS) [34], and induced hyperfiltration [35]; ingesting an equal amount of plant protein does not promote the same effects [36]. Most of the food within plant-based diets come from plant sources [37,38]. These types of diets are generally lower in protein and saturated fat, contain higher levels of potassium and phosphorus, are richer in fiber, and provide the body with additional nutrients in the form of vitamins, minerals, and phytochemicals. Adopting a plant-based diet has been shown to have numerous health benefits, such as a reduction in atherosclerotic plaque buildup, decreased risk of cardiovascular disease, decreased BMI, reduced body weight, and lower blood pressure [39,40], which are parameters that are clinically relevant for management of CKD patients [39].

Reductions in daily protein have produced some evidence in slowing CKD progression [41] by retarding the rate of kidney function decline [42]. However, determining an optimal amount of protein for CKD is complicated, especially when assessing the patient’s individual circumstances [43]. When considering a protein-restricted diet, the patient’s individual nutritional status should be evaluated with caution [41]. All previous protein-restricted diet studies are inconclusive [23,44].

### 2.3. Vitamin D

The primary role of vitamin D (VD) is to activate intestinal calcium reabsorption [45], but as kidney disease progresses, alterations in the biological mechanism occur. Low levels of active VD in ESRD patients are associated with increased bone reabsorption and reduced bone mineral density [46]. Studies report a progressive decline in VD of more than 80% from CKD 1–5, dialysis [47], and transplant patients [48]. Vitamin D metabolism is interrupted by the inability for the second hydroxylation step of 25-hydroxyvitamin D to occur, which converts it to the active form 1,25 dihydroxy vitamin D, which occurs in the kidneys [49]. Inhibition of 1,25 dihydroxy vitamin D induces hypocalcemia, which stimulates the parathyroid gland to release parathyroid hormone at persistent circulating levels [50,51]. Over time, this may result in renal osteodystrophy, including secondary parathyroidism, osteitis fibrosa, osteomalacia, and adynamic bone disease [45].

The current KDOQI guidelines for CKD nutrition state ergocalciferol or cholecalciferol effectively treats VD deficiency/inefficiency; however, specific dosing should be individualized and derived through a step-by-step approach [17]. This step-by-step approach includes monitoring 25(OH)D serum levels and serum calcium and serum phosphorus, which helps the healthcare team recommend specific dosing veered to the patient’s individual requirements [18]. Supplementation with ergocalciferol or cholecalciferol is essential in treating and preventing BMD disease in CKD [50,51]. A meta-analysis performed by Kandula et al. [52] suggests that supplementation of 1,25 dihydroxy vitamin D in CKD leads to increase in the serum levels and improves biochemical end-points. The study failed to observe any clinically significant outcomes due to observed improvements in biochemical end-points [52].

### 2.4. Calcium

Calcium balance is regulated by intestinal calcium absorption, kidney reabsorption, and calciotropic hormones that activate calcium exchange from the bone when serum calcium levels are low [18]. Insufficient calcium absorption and chronic calcium deficiency result in increased risk for hyperthyroidism and osteitis [17]. However, excessive calcium poses an increased risk for calcification, resulting in comorbidities and higher mortality [17]. Alterations in calcium metabolism are multifactorial and include the use of active vitamin D analogues. Research shows that ingesting about 800–1000 mg/d of calcium may be sufficient to maintain calcium balance for patients with CKD 3–4 in the absence of vitamin D analogues [17] (Table 2). However, calcium recommendations for early stages of CKD typically follow the RDA (1000–1200 mg/d) for adults because the level of kidney function has not yet disrupted calcium balance, although this is in individualized circumstances. Maintaining calcium balance is more complicated for CKD patients on dialysis, and hypercalcemia is relatively standard. Vitamin D is an important factor in maintaining calcium balance. VD supplementation therapy is prescribed to CKD patients with inefficient active VD levels to increase calcium reabsorption and prevent high serum para-thyroid hormone (PTH) and bone turnover [53]. Massart et al. [54] and Jean et al. [53] reported increased 1,25(OH)2D levels after cholecalciferol supplementation. Strong evidence shows the importance of adequate active VD for calcium balance, and it is achieved with VD supplementation in CKD patients [18].

### 2.5. Phosphorus

Phosphorus plays a critical role in bone formation, acid–base balance, and energy production [48]. The body’s ability to maintain phosphate balance is achieved by excreting excess phosphate in the urine. As CKD progresses, declining renal function prevents the kidneys from excreting enough phosphorus needed for phosphorus homeostasis [18]. The 2020 NKF guidelines recommended CKD 1–5 and HD patients receive an intake of phosphorus that keeps serum phosphorus levels within normal ranges (3.4–4.5 mg/dL) and to restrict dietary phosphate in the case of hyperphosphatemia [18,55] (Table 2). Hyperphosphatemia may lead to critical pathogenic consequences, including renal osteodystrophy, cardiovascular and soft tissue calcification, secondary hyperthyroidism, cardiac disease, and mortality in ESRD patients [56]. Phosphorus requirements depend on the stage of renal failure combined with the consideration to not restrict phosphorus intake to the point of malnutrition, which is mainly relevant to HD patients [57]. Despite the KDOQI revision for phosphorus intake in CKD, nephrologists recommend a phosphorus restriction of 800–1000 mg/d [10]; however, adequate studies are lacking that demonstrate the efficacy of 800–1000 mg dietary phosphorus restriction and the outcomes in CKD patients [18].

The three sources of dietary phosphorus are organic phosphorus from plant foods (bioavailability 20–40%), organic phosphorus from animal protein (bioavailability 40–60%), and inorganic phosphorus found in additives and processed foods (bioavailability ≈100%) [58]. Humans lack phytase, which is the enzyme that degrades phytates in plant foods, and this is why the bioavailability is the lowest of the three sources [58]. Inorganic phosphorus (additives) is almost entirely absorbed and may add up to 1000 mg/d of phosphorus from additives alone [26]. Choosing phosphorus-containing foods lower in bioavailability and without phosphate additives is recommended [17]. A study by Moe et al. that included CKD-4 patients reported lower phosphate levels in patients fed a 7-day vegetarian diet than patients fed a 7-day animal-based diet [25]. About 100 mg of phosphorus is found in 100 mL of milk and >500 mg per 100 g of cheese; the content of phosphorus is high in dairy products [59]. One study reported higher dietary phosphorus intake and a higher phosphorus to protein ratio in HD patient’s diets was associated with increased mortality risk in the preceding years, even after adjusting for phosphate binders [60]. Sources containing only organic phosphorus are more nutrient-dense than foods with phosphate additives, which are usually processed and high in sodium [30].

**Table 2:** Daily requirements for electrolytes in chronic kidney disease (CKD) patients.

Electrolytes	Damage in CKD	Recommendation	Outcome	Ref
Total calcium CKD 3–4 w/no use of taking active vitamin D analogues	Ca2+ deficiency ↑ risk secondary hyperparathyroidism and bone disorders. Excessive Ca2+ ↑ risk extraosseous calcification and CVD	800–1000 mg/day	Maintain Ca2+ balance	[18,29,61]
CKD 5 w/use of active vitamin D analogues	Ca2+ deficiency ↑ risk secondary hyperparathyroidism and bone disorders. Excessive Ca2+ ↑ risk extraosseous calcification and CVD	Individualize Ca2+ restriction based on the use of vitamin D analogues	Maintain Ca2+ balance and prevent hypercalcemeia	[18,29,62]
Dietary Phosphorus * CKD 1–5	High dietary phosphorus intake associated w/ accelerated progression of disease and greater 5-year mortality risk	adjust dietary phosphorus intake to maintain normal serum phosphate levels between 3.4–4.5 mg/dL	Maintain Ca2+ and PTH balance. ↓ Secondary hyperparathyroidism mineral and bone disorders. Slow progression of CKD	[29,63]
Dietary Potassium CKD1–5 or post-transplantation	Hyper/hypokalemia associated w/muscular weakness, hypertension, ventricular arrhythmias, and death. Hypokalemia associated w/peripheral neuropathy.	adjust dietary K+ intake to maintain serum potassium within 3.5–5.5 mEq/L	Slow progression of CKD. Prevention of peripheral neuropathy and other nerve related dysfunction.	[29,64,65]
Sodium (Na+) CKD 1–5 or post transplantation	↑ BP excessive fluid retention/increased weight	<2300 mg/day	↓ BP and normalize fluid balance/weight reduction/may ↓ proteinuria	[29,66,67,68]

* Phosphate recommendations recently changed; previously 800 mg, ↑ increased/high, ↓: decreased/lowered.

### 2.6. Potassium

Potassium (K) is the most abundant intracellular ion with a concentration of about 98%; it has many biological functions such as cellular metabolism and acid–base homeostasis [69]. It is also vital for cardiac function, neural transmission, muscular contractions, and glucose metabolism [67,70]. If potassium balance is disrupted by increased serum potassium, the patient is at risk for developing hyperkalemia (Table 2). Hyperkalemia is a severe metabolic condition that is often experienced in patients with CKD. The kidneys’ ability to excrete potassium is inversely related to a GFR function [69]. Hyperkalemia alters the nervous system’s function, causing electrophysiological dysfunctions [64,71], presenting clinical manifestations such as muscle weakness, paresthesia, paralysis, nausea, hypotension, cardiac arrhythmias, and cardiac arrest [67,70]. As CKD progresses, potassium levels are monitored closely; patients are advised to limit dietary potassium intake to maintain serum potassium levels within normal range (3.5–5.5 mEq/L) [17]. Potassium is rich in many foods such as vegetables, dark leafy greens, potatoes, tomatoes, fruit, coffee and tea, and citrus. CKD nutrition therapy recommends vegetables and fruits that are low in potassium and high in fiber along with [17] other nutrients, and to boil vegetables to decrease potassium concentration [17].

The ideal potassium intake is difficult to determine because of factors that influence serum potassium levels, such as medications, hydration level, acid–base status, glycemic control, adrenal function, and gastrointestinal complications [17]. It is essential to consider these factors when assessing the appropriate intake of potassium for a CKD patient, as the recommendations for potassium are individualized based on other preexisting health conditions the patient might have or be at risk for. The DASH diet is widely used as nutrition therapy for hypertension because of its effectiveness in lowering blood pressure, preventing and managing hypertension, and reducing cardiovascular risk [72]. The DASH diet may be protective against the progression of CKD, but its effectiveness in delaying the progression of the disease in CKD patients has not been established [72]. The DASH diet is high in potassium and low in sodium; it suggests four to five servings of fruits and vegetables a day, which sums up to about 4700 mg/d of potassium [72]. Studies on the DASH diet with CKD patients are scarce, and the few existing studies include CKD patients with serum potassium levels in normal range at the start of the study [62]. This is a limitation of the study for determining the efficacy of the DASH diet for CKD patients [73]. Another diet currently being studied for its benefits in CKD is the Mediterranean Diet (MedDiet). Instead of its focus being on low sodium and high potassium, it focuses on healthy fats, lean meats, and plant-based foods, which naturally offer a diet low in sodium. MedDiet studies began in the 1960s, and since then, increasing evidence supports the MediDiet to be protective against CKD and DM [51]. The MedDiet is rich in plant-based foods and low in processed and red meat [74]. It is moderate in seafood, eggs, dairy, and red wine; and olive oil is the main source of added fat [75]. Adherence to the MedDiet helps prevent and manage CVD and DM [71,76], which would in turn help prevent CKD. However, the role of the MedDiet in delaying CKD progression remains uncertain due to insufficient data on patients with pre-existing CKD or dialysis [70].

Additionally, Kalemic control is further compounded by extensive use of the renin–angiotensin–aldosterone system inhibitor (RAASI) therapy in CKD patients [77]. Development of hyperkalemia in CKD patients requires lowering the dose or discontinuation of the RAASI therapy to protect patients from developing cardiovascular events and end stage kidney disease.

The true benefit of potassium restriction in CKD is not clear, considering that a diet with a high content of potassium-rich foods, such as plant-based low-protein diets, can be as beneficial on the prognosis. Potassium levels in serum can further be improved using the new K-binders, whose benefits and efficacy are shown in randomized control trials [78,79], allowing implementing plant-based low-protein diets with lower risk of hyperkalemia. Further research investigating the effect of a low-potassium diet and the progression of renal disease are required. It is unclear whether a potassium-restricted diet can slow CKD progression; however, research shows that it may reduce all-cause mortality in CKD [79].

### 2.7. Sodium

Sodium overload in advanced CKD patients induces extracellular volume, which may lead to hypertension and heart failure. Hypertension is a known risk factor for the progression and mortality of CVD; however, the effect of sodium on the advancement of CKD remains inconclusive [18]. A recent working hypothesis suggests that the accumulation of sodium in interstitial space induces inflammatory toxicity that is independent of volume, and it is mediated by immune cells [80]. Sodium accumulation in the body increases as the GFR declines over time [81].

A low sodium diet is central to the management of hydro-saline homeostasis, reducing systolic and diastolic blood pressure as well as proteinuria [82]. Nevertheless, a low-salt diet must be carefully monitored in older patients, considering they are at higher risk for acute kidney injury and damaged renal autoregulation [51]. The efficacy of low sodium intake and the reduction in BP in hypertensive patients dates to 1948 [83,84], currently reaching a worldwide understanding of the relationship between sodium and hypertension [85,86]. Patients with hypertension have a 75% increased risk of developing CKD than normotensive individuals [83,87] and a 25% increased risk of developing a decline in GFR among pre-hypertension patients [84,87]. The McMahon et al. study assessed the effects of high- vs. low-sodium diets on BP, 24 h protein and albumin excretion, and fluid status in 20 hypertensive stage 3–4 CKD adult patients [66]. The study concluded that the low-sodium diet resulted in statistical and clinically significant declines in BP, extracellular fluid volume, albuminuria, and proteinuria in study patients [66].

Nonetheless, sodium restriction is protective for the onset of hypertension. There is plentiful and strong evidence in the efficacy to prescribing a sodium-restricted diet for disease management in CKD [85]. For CKD stages 3–5, the most recent sodium intake recommendation is a maximum of 2.3 g/d and to make sodium restriction a lifestyle for controlling fluid volume and maintaining a desirable weight for CKD 3–5D [17] (Table 2). Effective habits to reduce sodium may be achieved by identifying high-sodium foods such as processed foods, canned vegetables, pickled and fermented foods, soups, chips, salted nuts and seeds, processed foods, and restaurant items. Simple modifications such as choosing unprocessed foods, choosing frozen over canned vegetables, avoiding soups and pickled and fermented foods, choosing unsalted nuts and seeds, and requesting no additional salt when ordering out are beneficial for achieving a restricted sodium diet.

### 2.8. Whole Food Plant-Based Diet

Studies report that a whole food plant-based (WFPB) diet reduces the risk for T2DM and CVD in CKD patients [2]. A WFPB diet is more restrictive than a vegan diet by the exclusion of processed and refined foods such as isolated vegetable oils, bleached flours, and cane and beet root sugar; the diet focuses on increased fiber and nutrient-dense foods that are low in protein and energy [2]. Whole grains, nuts, seeds, legumes, monosaturated oils, fruits and vegetables, and tubers make up the foods in a WFPB diet [88,89].

WFPB diets provide about 75% of carbohydrates (CHO), emphasizing dietary fiber [90,91]. Fiber intake of about 27 g/d reduces serum urea and creatine in CKD; high serum urea and creatine indicate abnormal GFR [90,91]. High fiber intake shifts the gut microbiota by increasing the amount of gut microflora that break down and process fiber [92]. Soluble fiber intake such as apples and oats reduces serum cholesterol, postprandial glucose, insulin response [92,93], and induce satiety from delayed gastric emptying [93,94]. Insoluble fiber such as whole grains and legumes increase motility and transit time by softening stool and promoting regular bowel movement, which is especially critical for CVD and CKD patients as they commonly experience slowed colonic transit time [92,93]. WFPB diets are significantly higher in fiber than other diets resulting in several health benefits for just fiber alone [65,92].

WFPB diets do not restrict fat intake; however, the foods promoted are made up of monounsaturated and polyunsaturated fats and limit processed oils and saturated fat [2]. Previous studies show a daily caloric intake of total fat to be less than 15% in WFPB diets, which is protective against CVD [91]. It is well established that omega-3 fatty acids reduce inflammation [94,95], blood pressure, and increase HDL cholesterol [95,96]. Plant-based omega-3s are in foods such as flaxseeds, chia seeds, walnuts, olives, and some dark green vegetables [2]. The consumption of 1.5–3 g/d of omega-3s is associated with CVD prevention in CKD patients [94].

It is challenging for patients to comply with a restricted phosphorus diet because it is found in most foods [60]. Many fruits and vegetables contain a slight phosphorus trace, while its content is higher in seeds, nuts, and legumes; and it is even higher in animal products [68]. However, plant foods contain phytates that limit phosphorus’s gastrointestinal absorption, decreasing the bioavailability of phytate-based phosphorus [68]. Additionally, a WFPB diet restricts processed foods and sugar, including restructured meat and soft drinks, which contain inorganic phosphorus-based additives for preservation. These additives generally go unnoticed due to their complex and unrecognizable names, with inorganic phosphorus having the highest absorption rate, at more than 90% [97,98].

A WFPB diet is naturally low in sodium due to the restriction of processed foods, assisting the patient with maintaining appropriate sodium levels. Additionally, WFPB diets are generally lower in energy and may be beneficial for weight management. However, caution and careful planning are critical to avoid inadequate energy intake and PEW, which could worsen the patient’s health, increasing their risk for morbidity and mortality [57]. A wide variety of plant-based foods need to make up the diet and increased consumption of starchy vegetables, fruits, and legumes to meet the RDAs for protein and energy [2]. A drawback in the WFPB diet is the need to supplement with vitamin B12, because sufficient vitamin B12 intake is only met through the consumption of animal-based foods [2]. Although evidence is growing that supports the positive health benefits of WFPB diets, there is a need for more research to determine any nutritional deficiencies or other adverse health effects from a WFPB diet in a clinical population with CKD patients [2].

## 3. The Role of a Registered Dietitian

Dietary education and patient counseling provided by a registered dietitian (RD) is essential for preventing and managing CKD. Careful and detailed dietary planning, frequent assessment of nutritional status, and dietary monitoring compliance are critical for successful dietary management.

The progressive decline in GFR is a risk factor for the development of metabolic acidosis. The main goal of therapy is to prevent or correct this metabolic acidosis, which has been shown to slow down the progression of CKD to end-stage renal disease [99]. The biggest contributor to this acid pool is the consumption of a diet higher in animal proteins [100]. The simplest treatment for this metabolic acidosis includes dietary management by reducing the protein in the diet or switching the diet to an increase in plant-based proteins [101]. It has been shown that dietary intervention of lowering protein intake or switching to plant-based protein reduces metabolic acidosis in stage 3–4 CKD patients [63].

Primary and secondary studies out of the MDRD study suggest that dietary interventions such as a low-protein diet reduce the rate of kidney function decline and lower the risk of ESKD in CKD patients [13,102]. Dietary interventions, such as low-protein diet, have been shown to retard the progression of CKD [102]. The dietary restriction of protein and phosphorous are shown to reduce the decline of kidney function and has been observed in type 1 diabetes patients [103]. The consensus among clinicians is that dietary interventions slow the rate of kidney function and potentially reduce the risk of end-stage kidney disease in patients with diabetes and CKD.

CKD patients often have or are at risk for comorbidities that entail specific diet management recommendations; this can be challenging and overwhelming. Additionally, CKD diet recommendations alter depending on the disease stage; this can create confusion for the patient. The dietitian has a more significant role than just providing dietary advice and recommendations for the patient. Counseling should be individualized and altered to the patient’s overall health, pre-existing conditions, and personal preferences. Adopting and adhering to a new diet requires the ability to motivate and inspire patients to make changes that will improve their health and prevent morbidities, although the changes may be uncomfortable for the patient. Adequate education about the rationale of the recommendations and how the patient will benefit are essential to convey. Equally as important is to assess the retention and understanding of the patient from the nutrition education. Through a thorough patient assessment and evaluation, the dietician may help prevent kidney disease by carefully monitoring their diabetic, hypertensive, and CVD patients by ordering the appropriate screening labs. It is imperative to regularly screen the patient for CVD, T2DM, malnutrition, and anemia, as they are at high risk of developing them. Providing alternative food options tailored to the patient’s likes and dislikes to replace restricted foods is more productive than focusing on the restrictions. Providing substitution education to the patient is essential to attain and maintain patient compliance and achieve successful dietary management.

The major limitation of this review is that although it is a literature review, we did not performed a systematic review of the literature. The literature survey was performed for a narrative review of the currently available studies to attempt to compile available studies under this review. To keep the review within limits, the search strategy was not comprehensive, and the studies were not assessed critically. Furthermore, this review was limited to protein, calcium, phosphate, and VD and electrolytic balance; it does not provide more comprehensive information on the clinical management of CKD.

## 4. Future Research and Clinical Practice

Secondary analysis of the MDRD study showed that patients with low protein intake during follow-up began experiencing uremic symptoms at lower GFR than patients with higher protein intake [103]. The reduced risk of end-stage renal failure reported may be from a delay in starting dialysis due to improved uremic symptoms rather than delayed kidney decline [103]. In addition, the study included 200 (24%) polycystic kidney disease (PKD) patients who may have contributed to data showing a delay in renal dysfunction due to the differences in the course of disease progression between CKD and PKD [103]. The INTERMAP Study lacked the use of “gold standard” diet assessments, food variation among different countries, and variation in dietary intake, which weakens the associations between nutrient intake and blood pressure [33].

Despite the large number of clinical trials being performed in the clinical and nutritional management of CKD, very few of these have translocated into clinical practices due to the lack of strong associations, not so clear research design, or low number of study subjects. There is a demand for future research to provide conclusive information that will assist clinicians and dietitians to make the most appropriate recommendations for their patients. Evaluating the impact of MNT on CKD progression by analysis of associated risk factors in patients with comorbidities is needed [18]. The clarity regarding which stage of CKD is most appropriate to alter protein intake is necessary. Future VD studies are required to determine the correct dosing and type of VD supplements for CKD patients. Future studies examining, comparing, and contrasting WFPBD, Mediterranean diet, and DASH diet in CKD patients to determine their effects on clinical outcomes are needed. Another challenge in CKD patients is not following the dietary recommendations. Research should be focus on boosting patient diet compliance by developing methods that will improve compliance and long-term adherence to nutrition prescriptions.

## 5. Conclusions

Chronic kidney disease is a growing health crisis in the U.S. Diabetes and hypertension are the leading causes of CKD development; as the US is experiencing an increasing prevalence of both, CKD is expected to remain a critical national health issue. At ESRD, the kidneys have lost their ability function, and as a result, a series of malfunctions occur that lead to adverse health problems and health outcomes. Once diagnosed with ESRD, the patient either will be on dialysis for the rest of their life or receive a kidney transplant. Medical nutrition therapy with a RD is a critical aspect in the intervention for CKD because it is almost solely through nutrition that aids in the delay of the disease’s progression and the prevention of comorbidities and mortality. 

## Figures and Tables

**Table 1:** Protein and energy requirements and recommendations for adult chronic kidney disease (CKD) patients.

Nutrients	Damage in CKD	Recommendation	Outcome	Ref
Protein CKD 3–5 patients. Not on Dialysis/Without Diabetes	Proteinuria/glomerular sclerosis/hyperfiltration/intraglomerular hypertension and hyperperfusion	0.55–0.6 g/kg body wt/day	Reduce uremia, uremic toxins, and hyperfiltration Improve organ function and renal hemodynamics	[18,21,22]
CKD 3–5 Pts Not on Dialysis and w/Diabetes	Proteinuria/glomerular sclerosis/hyperfiltration/intraglomerular hypertension and hyperperfusion	0.6–0.8 g/kg body wt/day	Reduce uremia, uremic toxins, and hyperfiltration Improve organ function, and renal hemodynamics	[18,25,26]
HD and PD Pts w/ and without Diabetes	Proteinuria/glomerular sclerosis/hyperfiltration/intraglomerular hypertension and hyperperfusion	1.0–1.2 g/kg body wt/day	Reduce uremia, uremic toxins, and hyperfiltration Improve organ function, and renal hemodynamics.	[18,25,26]
Energy Intake CKD 1–5D or post transplantation	Inadequate intake ↑ risk PEW, ↑ risk malnutrition. Excessive intake ↑ risk CVD, ↑ risk diabetes	25–35 kcals/kg body wt/day	Maintain neutral nitrogen balance and body composition.	[18,28]

Recommendations are for metabolically stable patients under strict clinical supervision. ↑ increased/high.

## Data Availability

Not applicable.
